# Estimating the referral rate for cancer genetic assessment from a systematic review of the evidence

**DOI:** 10.1038/sj.bjc.6603432

**Published:** 2007-01-23

**Authors:** C Featherstone, A Colley, K Tucker, J Kirk, M B Barton

**Affiliations:** 1 Department of Oncology, Beatson Oncology Centre, Glasgow, G11 6NT, Scotland; 2 Department of Clinical Genetics, Liverpool Hospital, Sydney, Australia; 3 Department of Genetics, Prince of Wales Hospital, Sydney, Australia; 4 Department of Genetics, Familial Cancer Service, Westmead Hospital, Sydney, Australia; 5 Collaboration for Cancer Outcomes Research and Evaluation (CCORE), Liverpool Health Service, Liverpool Hospital, Sydney, Australia

**Keywords:** genetic assessment, systematic review, cancer genetics

## Abstract

To estimate the optimal proportion of new patients diagnosed with cancer who require assessment and evaluation for familial cancer genetic risk, based on the best evidence available. We identified evidence of the patients who require assessment for familial genetic risk when diagnosed with cancer through extensive literature reviews and searches of guidelines. Epidemiological data on the distribution of cancer type, presence of a family history, age and other factors that influence referral for genetic assessment were identified. Decision trees were constructed to merge the evidence-based recommendations with the epidemiological data to calculate the optimal proportion of patients who should be referred. We identified ‘high probability’ and ‘moderate probability’ groups for having a genetic susceptibility. The proportion of patients diagnosed with cancer in Australia who have a high probability of having a genetic predisposition and who should be referred for genetic assessment is 1%. If the moderate probability group is also assessed this proportion increases to 6%. This model has identified the proportion of new patients diagnosed with cancer who should be referred for genetic assessment. This data is the first step in determining the resources required for provision of an adequate cancer genetic service.

Major advances in the understanding of the molecular basis of cancer have implications for all aspects of cancer management, including prevention, screening and treatment. Increasing knowledge about familial cancer syndromes (Lindor *et al*, 1998), and the ability to detect specific germline mutations in various cancer-associated genes (Fearon, 1997) means that cancer genetics should be included in standard clinical care. Estimates for which there is a familial predisposition range from 5 to 10% (Fearon, 1997; Lindor *et al*, 1998).

Although there are tools available to enable doctors and patients to estimate cancer risk and prognosis these cannot replace comprehensive cancer genetics clinics that offer other services to individuals who are concerned about their family history of cancer. These services include risk assessment and education, facilitation of genetic testing, pre- and post-test counselling, provision of personally tailored cancer risk management options and recommendations, and psychosocial counselling and support services (Pichert, 2004). The cost effectiveness of genetic testing is influenced by targeting genetic services to patients with a strong family history of cancer rather than screening the entire population (Griffith *et al*, 2004)

In Australia, the National Health and Medical Research Council (NHMRC) have published guidelines on the familial aspects of cancer, recommending that patients with an above-average risk contributing to the development of their cancer should be referred to a cancer genetics clinic for further assessment (NHMRC, 1999a). These ‘at-risk’ individuals are identified through index relatives who have been diagnosed with cancer.

The objectives of this study are to estimate the ideal proportion of new cancer cases that should be referred for genetic assessment from the best available evidence. This will identify the number of index cases overall and for each tumour type that requires assessment by a cancer genetics service. The model can be modified to incorporate new indications for referral as molecular tests are found or when appropriate prevention strategies are identified.

## MATERIALS AND METHODS

### Optimal referral rate

We identified from guidelines the indications for a patient diagnosed with cancer who requires referral to a cancer genetics clinic for further assessment, counselling or testing. We also identified epidemiological evidence for the different attributes associated with probability of a genetic component of cancer risk. We used Australian data wherever possible so that the result would be relevant for local service planning. The model does not include childhood or rare cancers that account for less than 1% of all cancer cases.

### Indications for genetic counselling

In this study, an indication for genetic counselling was defined as a clinical situation where a hereditary trait is likely to be the primary cause of the cancer. Identification of this trait would have an effect on the overall clinical outcome, either for the patient or their family members. For genetic counselling to be indicated there must be a diagnostic genetic test available, benefit from screening or early detection or an appropriate early intervention available to influence cancer risk and/or survival.

In Australia, the NHMRC have published guidelines on the familial aspects of cancer, recommending that patients with an above-average risk contributing to the development of their cancer should be referred to a cancer genetics clinic for further assessment (NHMRC, 1999a). Guidance on indications that can be used to help stratify the potential level of risk (high, moderate and average) are included.

Four groups were identified:

1. *High probability*: where the likelihood that a hereditary trait is the cause of the cancer is substantially greater than average. This group includes people whose lifetime risk for breast cancer is likely to be between 25 and 50%, those who meet the modified Amsterdam criteria for Hereditary non-polyposis colorectal cancer and those at potentially high risk of developing ovarian cancer (NHMRC, 1999b).
2. *Moderate probability*: where the likelihood that a hereditary trait is the cause of the cancer is greater than average. This group includes people whose lifetime risk for breast cancer is likely to be between 12 and 25%, and those who are likely to have a relative risk of colorectal cancer that is three- to six-fold.
3. *Low probability*: where the likelihood that a hereditary trait is the cause of the cancer is no higher than in the general population.
4. *Research*: a group where a genetic predisposition may have contributed to the development of cancer but where currently no diagnostic genetic test is available, or no benefit from screening or early detection has been determined or there is no appropriate early intervention available to influence cancer risk and/or survival.

### Search strategy and selection criteria

We searched the National Guidelines Clearinghouse, Medline and the major cancer services that have guidelines on the internet for national level guidelines and guidelines issued by major institutions on the indications for referral to a familial genetics service.

There are Australian NHMRC clinical practice guidelines on familial aspects of cancer, colorectal cancer, breast cancer and melanoma. Two American sources of guidelines identified were the comprehensive Physicians Data Query (PDQ) database of the US National Comprehensive Cancer Institute (NCI), and the National Comprehensive Cancer Network (NCCN). The level of evidence that supported each recommendation for use of genetic assessment was classified using the Australian NHMRC hierarchy of levels of evidence.

The optimal genetic assessment utilisation tree was constructed using TREEAGE DATA™ software (version 3.5). Each terminal branch represents either ‘referral for genetic assessment’ or ‘no referral for genetic assessment’ as the management decision. The proportion of patients requiring referral for genetic assessment was subdivided into patients who should be categorised as having either a high or moderate probability of hereditary cancer.

Each branch of the tree signifies an attribute that affects a management decision (e.g. other family member with cancer). Above each branch is a description of the specific attribute that has led to that decision. Each number below the branch signifies the proportions of the attribute based on epidemiological data.

### Epidemiological data

The source with the highest ranking was used to determine the incidence of each indication for genetic assessment. We used Australian national and state cancer registry epidemiological data wherever available to make the results of this study relevant for planning future cancer genetic services in Australia. When national data were unavailable, more specific data sets were used according to a published hierarchy. Epidemiological data were ranked according to the schema in Table 1.

## RESULTS

**Figure 1:**
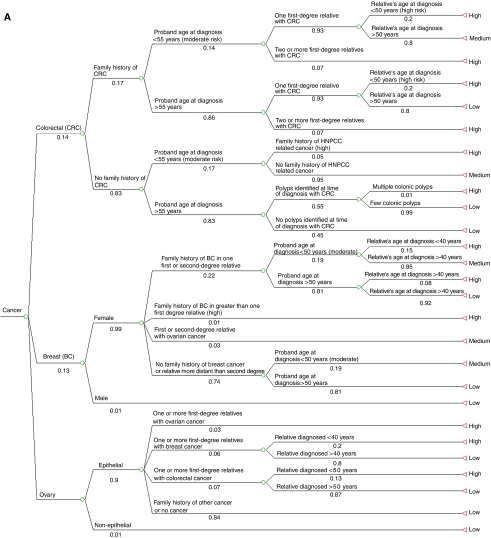

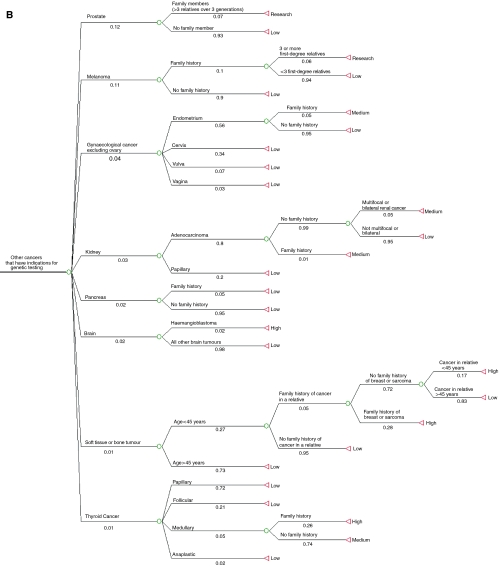
(**A**) Optimal referral for genetic assessment tree for colorectal, breast and ovarian cancer. (**B**) Optimal referral for genetic assessment tree for other cancers.

The clinical situations for which referral for genetic assessment is recommended, and the guideline or source of evidence for the recommendation are tabulated (Tables 2 and 3). In Table 2, the outcome numbers correspond to the outcome positions in the tree (Figure 1A and B). The last column represents the incidence of each clinical indication for referral for genetic assessment as a proportion of patients diagnosed with cancer. Table 3 show the epidemiological data corresponding to each branch point, and the source of the data, as well as the hierarchical level of the data, obtained.

Each branch of the tree (Figure 1A and B) signifies an attribute that affects a management decision. Above each branch is a description of the specific attribute that has led to the treatment decision. Each number below the branch signifies the proportions of the attribute based on epidemiological data. Each terminal branch of the tree showed whether or not referral for genetic assessment was recommended for patients with those particular attributes.

### Outcome

There were possible 68 ‘outcomes’ for this tree of which 16 recommended that patients had a high probability of familial cancer and should be considered for referral to a cancer genetics service. A further nine ‘outcomes’ classified patients as at least moderate risk. The optimal proportion of patients diagnosed with cancer who should be referred for genetic assessment for high or moderate risk was calculated to be 0.06, that is, 6% of all patients diagnosed with cancer in Australia should be referred for genetic assessment, based upon the best available evidence. The optimal proportion of ‘high-probability’ patients is 1%.

Referral for cancer genetic assessment has the greatest impact for patients diagnosed with breast, colorectal and ovarian cancer. The proportion of patients diagnosed with each of these cancers who are at moderate or high probability are shown in Table 4 and Figure 1A.

Prostate cancer and melanoma fall into the group that is of research interest at present. The proportions of patients for whom genetic assessment may be eventually indicated were 7 and 1%, respectively.

## DISCUSSION

All oncology providers must be aware of the hallmarks of susceptibility to hereditary cancer in order to appropriately identify patients who might benefit from comprehensive cancer genetic counselling. Guidelines on patients who are at risk have been published by national groups (Trepanier *et al*, 2004) to help identify patients who require referral. Variations in referral may result from both lack of proper identification of an ‘at risk’ individual, clinician's nihilism in what can be achieved by screening or earlier diagnosis or variations in accessibility to these clinics (McDonald *et al*, 2004).

Both International and National guidelines and data were used to complete this study, and it is therefore thought to be broadly applicable. Australian cancer registry data were used for the initial proportions of each cancer type. This is recorded in the first column of Table 4. The model can be adapted for regional services planning by incorporating data from national or local cancer registries. This study not only identifies the overall proportion of patients who might benefit from referral, but also the proportion by each cancer type. These models can be used to identify who should be considered for referral, dependent on patients' wishes and relatives who might benefit from the information obtained.

This model identifies the probability for newly diagnosed cancer patients for whom an inherited trait might be identified, and early intervention or screening may be warranted. The model does not include childhood or rare cancers such as phaeochromocytoma, although there is emerging evidence of a genetic predisposition (Neumann *et al*, 2002), or retinoblastoma, where all new cases should be referred for genetic assessment.

Currently in Australia only patients who are at high risk are eligible for referral. However, an optimal service should include referral of patients who are at high and moderate probability of having a genetic susceptibility, where confirmation of the family history and more detailed assessment of their probability of genetic predisposition can be determined by genetic counsellors and specialists.

Comprehensive reviews of both the psychological impact and an economic evaluation of testing and counselling have been performed (Braithwaite *et al*, 2004; Griffith *et al*, 2004). Genetic counselling for familial cancer is associated with an improvement in knowledge but does not have an adverse effect on affective outcomes.

This model identifies the optimal number requiring counselling if all index cases have a single relative who wishes to benefit from this service. It starts with the index case rather than the unaffected person concerned about their family history. For each index case data on family members is required with the number requiring referral ranging from no family members to several. This data is not currently available but may be obtainable from local databases for some of the commoner malignancies.

From the optimal proportion identified in this study, further work is required to determine the actual number of patients diagnosed with cancer in each tumour site that require access to this service, and the number of relatives that may require assessment when a genetic predisposition is identified. This can be included in the economic evaluation to help determine the value of this service to individuals, families and society. Work is also required to assess why patients may not be referred to a cancer genetic service, which is likely to include lack of awareness of the benefits of counselling and testing, lack of access to a service, patient refusal, no relatives or no family members residing in the country, and referring doctors' uncertainty as to the benefits of the service.

## CONCLUSIONS

The planning of efficient cancer genetics services for a population requires a rationale estimate of its need. This model identifies the proportion of patients with cancer who may benefit from such a service by using an evidence-based approach and is the first step to appropriate planning and resourcing of cancer genetics services. Further research is required to identify both the actual proportion of the population who are referred to a cancer genetics service and also reasons why patients are currently not referred.

## Figures and Tables

**Table 1:** Hierarchy of epidemiological data

**Quality of source**	**Source type**
*α*	Australian National Epidemiological data
*β*	Australian State Cancer Registry
*γ*	Epidemiological databases from other large international groups (e.g. SEER)
E	Results from reports of a random sample from a population
*ε*	Comprehensive multi-institutional database
Z	Comprehensive single-institutional database
Θ	Multi-institutional reports on selected groups (e.g. multi-institutional clinical trials)
*λ*	Single-institutional reports on selected groups of cases

**Table 2:** Genetics: indications for genetic assessment: levels and sources of evidence

**Outcome no.**	**Clinical scenario indicated**	**Level of evidence**	**Reference**	**Proportion of patients with cancer (high)**	**Proportion of patients with cancer (high and moderate)**
1	CRC, family history, age one first-degree relative with CRC age of relative<50 years<55 years,	III	CCV (2004); NHMRC (1999b)	0.001	0.001
2	CRC, family history, age <55 years, >one first-degree relative with CRC age of relative >50 years	III	CCV (2004); NHMRC (1999b)	<0.001	0.002
3	CRC, family history, <55 years 2 or more first-degree relatives with CRC	III	CCV (2004); NHMRC (1999b)	<0.001	<0.001
4	CRC, family history, age >55 years one first-degree relative with CRC age of relative<50 years	III	CCV (2004); NHMRC (1999b)	0.004	0.004
6	CRC, family history, <55 years 2 or more first-degree relatives with CRC	III	CCV (2004); NHMRC (1999b)	0.001	0.001
7	CRC, no family history of CRC age <55 years, history of HNPCC related cancer	III	CCV (2004); NHMRC (1999b)	0.001	0.001
8	CRC, no family history of CRC age <55 years, no family history of HNPCC related cancer	III	CCV (2004); NHMRC (1999b)	<0.001	0.02
9	CRC, no family history of CRC age >55 years, polyps identified, multiple polyps	III	CCV (2004); NHMRC (1999b)	0.001	0.001
12	Breast, female, family history, <50, relative <40 years	III	NCCN (2004)	0.001	0.001
13	Breast, female, family history <50, relative >40 years	III	NCCN (2004)	<0.001	0.006
14	Breast, female, family history >50, relative <40 years	III	NCCN (2004)	0.002	0.002
16	Breast, female, >one relative	III	CCV (2004)	0.001	0.001
17	Breast, female, family history of ovarian cancer	III	CCV (2004)	<0.001	0.004
18	Breast, female, no family history age <50 years	III	CCV (2004)	<0.001	0.018
21	Ovarian cancer, epithelial, family history	III	NHMRC (1999a); CCV (2004)	<0.001	<0.001
22	Ovary, epithelial, one or more relatives with breast cancer, relative <40 years	III	NHMRC (1999a); CCV (2004)	<0.001	<0.001
24	Ovary, epithelial, one or more relatives with colorectal cancer relative <50 years	III	NHMRC (1999a); CCV (2004)	<0.001	<0.001
33	Endometrium, family history	III	Smith *et al* (2002)	<0.001	0.001
38	Kidney cancer, adenocarcinoma no family history, age >50 years multifocal or bilateral tumours	III		<0.001	0.001
40	Kidney cancer, adenocarcinoma family history	III	CCV (2004)	<0.001	<0.001
44	Brain tumour haemangioblastoma	III	CCV (2004)	<0.001	<0.001
46	Soft tissue or bone tumour, age <45years, family history of cancer, no breast or sarcoma, relative <45	III	CCV (2004)	<0.001	0.001
48	Soft tissue or bone tumour, age <45years, family history of cancer, breast or sarcoma	III	CCV (2004)	<0.001	<0.001
53	Thyroid cancer, medullary, family history	III	NCI (2004)	<0.001	<0.001
54	Thyroid cancer, medullary, no family history	III	NCI (2004)	<0.001	<0.001

Total proportion of all patients with cancer who should be referred for genetic assessment 0.06 (6%).

^*^Level I, systematic review of all relevant randomised studies; level II, at least one properly conducted randomised trial; level III, well-designed randomised controlled trials without randomisation. Includes trials with pseudorandomisation or comparative studies; level IV, case series;

**Table 3:** Genetics: the incidence of attributes used to define indications for referral for genetic assessment

**Population of interest**	**Attribute**	**Proportion of population with attribute**	**Reference**	**Quality of information**
All registry cancers	Colorectal cancer	0.14	AIHW (2004)	*α*
Colorectal cancer	Family history	0.17	Fuchs *et al* (1994)	*γ*
CRC, family history	<55 years	0.38	Fuchs *et al* (1994)	*γ*
		0.14	St John *et al* (1993)	*λ*
CRC, family history, <55 years	Only one affected relative	0.93	St John *et al* (1993)	*λ*
CRC, family history, <55 years	Relative <50 years	0.21	St John *et al* (1993)	*λ*
Only one affected relative				
CRC, no family history	<55 years	0.17	Kune *et al* (1989)	*λ*
CRC, no family history,<55	Family history of HNPCC related cancer	0.05	Slattery and Kerber (1994)	ζ
CRC, no family history	Proportion of patients with polyps	0.55	Boutron *et al* (1997)	*λ*
>55 years,				
CRC, no family history	Proportion with multiple polyps	0.01	Muto *et al* (1975)	*ε*
>55, polyps			O'Brien *et al* (1990)	
All registry cancers	Breast cancer	0.13	AIHW (2004)	*α*
Breast Cancer	Female	0.99	AIHW (2004)	*α*
Breast cancer, female	Family history in one 1° or 2° relative	0.22	Slattery and Kerber (1993)	ζ
	Family history in >one 1° relative	0.01		
	Ovarian cancer in one 1° or 2° relative	0.03		
Breast cancer, female	Age <50years	0.23	Slattery and Kerber (1993)	ζ
family history of breast cancer				
Breast cancer, female	Relative <40years	0.15	CGHFBC (2001)	θ
family history of breast cancer,				
age <50years				
Breast cancer, female	Relative <40years	0.08	CGHFBC (2001)	θ
family history of breast cancer,				
age >50years				
Breast cancer, no family history	Age <50 years	0.19	Slattery and Kerber (1993)	ζ
All registry cancers	Ovarian cancer	0.01	AIHW (2004)	*α*
Ovarian cancer	Epithelial	0.9	AIHW (2004)	*α*
Ovarian cancer, epithelial	Relative with ovarian cancer	0.03	CGHFBC (2001)	*ε*
	Relative with breast cancer	0.06	Purdie *et al* (1995)	
	Relative with bowel cancer	0.05		
Ovarian cancer, epithelial	Relative <40 years	0.2	Easton *et al* (1996)	*γ*
Relative with breast cancer				
Ovarian cancer, epithelial	Relative <50 years	0.13	Easton *et al* (1996)	*γ*
Relative with bowel cancer				
All registry cancers	Prostate cancer	0.12	AIHW (2004)	*α*
Prostate cancer	>3 relatives over	0.07	Bratt *et al* (1999)	*ε*
	3 generations		Carter *et al* (1993)	
			Keetch *et al* (1995)	
All registry cancers	Melanoma	0.11	AIHW (2004)	*α*
Melanoma	Positive family history	0.1	Aitken *et al* (1994)	*β*
			Ang *et al* (1998)	
All registry cancers	Lung cancer	0.1	AIHW (2004)	*α*
All registry cancers	Gynaecological cancers	0.04	AIHW (2004)	*α*
	excluding ovary			
Gynaecological cancers	Endometrial cancer	0.56	AIHW (2004)	*α*
excluding ovary				
Gynaecological cancers	Cervix	0.34	AIHW (2004)	*α*
excluding ovary	Vulva	0.07		
	Vagina	0.03		
Endometrial cancer	Family history	0.05	Boltenberg *et al* (1990)	θ
			Gruber and Thompson (1996); Sandles *et al* (1992)	
All registry cancers	Head and neck cancer	0.04	AIHW (2004)	*α*
All registry cancers	Lymphoma	0.04	AIHW (2004)	*α*
All registry cancers	Unknown primary	0.04	AIHW (2004)	*α*
All registry cancers	Kidney cancer	0.03	AIHW (2004)	*α*
Kidney	Adenocarcinoma	0.8	AIHW (2004)	*α*
Kidney, adenocarcinoma	No family history	0.99	Czene and Hemminki (2002)	*γ*
Kidney, adenocarcinoma,	Multifocal or bilateral cancer	0.05	Neumann *et al* (1998)	*ε*
No family history				
All registry cancers	Bladder cancer	0.03	AIHW (2004)	*α*
All registry cancers	Leukaemia	0.03	AIHW (2004)	*α*
All registry cancers	Gastric cancer	0.02	AIHW (2004)	*α*
All registry cancers	Pancreatic cancer	0.02	AIHW (2004)	*α*
Pancreatic	No family history	0.95	Silverman *et al* (1999)	*ε*
All registry cancers	Brain tumour	0.02	AIHW (2004)	*α*
Brain tumour	Haemangioblastoma	0.02		*λ*
All registry cancers	Soft tissue and bone	0.01	AIHW (2004)	*α*
Soft tissue and bone	<45 years	0.3	AIHW (2004)	*α*
Soft tissue and bone,	Family history of cancer	0.05		
<45 years				
Soft tissue and bone	Family history of breast or sarcoma	0.28	Moutou *et al* (1996)	*λ*
<45 years, family history				
of cancer				
Soft tissue and bone	Cancer in relative <45 years	0.17	Moutou *et al* (1996)	*λ*
<45 years,no family history				
of cancer				
All registry cancers	Thyroid	0.01	AIHW (2004)	*α*
Thyroid cancer	Papillary	0.72	Hundahl *et al* (1998)	*γ*
	Follicular	0.21		
	Medullary	0.05		
	Anaplastic	0.02		
Medullary thyroid cancer	Familiy history	0.26	Moley *et al* (1998)	*λ*
All registry cancers	Liver cancer	0.01	AIHW (2004)	*α*
All registry cancers	Oesophagus	0.01	AIHW (2004)	*α*
All registry cancers	Gallbladder cancer	0.01	AIHW (2004)	*α*
All registry cancers	Myeloma	0.01	AIHW (2004)	*α*
All registry cancers	Testicular cancer	0.01	AIHW (2004)	*α*
All registry cancers	Other	0.01	AIHW (2004)	*α*

**Table 4:** Proportion of patients who require assessment by tumour site and probability

**Tumour type**	**Proportion of new cancers in Australia**	**Role for genetics**	**High %**	**High and moderate %**	**Moderate %**	**Low %**	**Research %**
Colorectal	14	Yes	5	21	16	79	
Breast	13	Yes	3	24	21	76	
Ovary	1	Yes	5	5	0	95	
Gynae-ovary	4	Yes	0	3	3	97	
Kidney	3	Yes	0	5	5	95	
Brain	2	Yes	2	2	0	100	
Sarcoma	1	Yes	1	1	0	99	
Thyroid	1	Yes	1	5	4	95	
Lung	10	No	0	0	0	100	
Prostate	12	Yes	0	0	7	93	7
Melanoma	11	Yes	0	0	1	99	1
Head and neck	4	No	0	0	0	100	
Lymphoma	4	No	0	0	0	100	
UK primary	4	No	0	0	0	100	
Bladder	3	No	0	0	0	100	
Leukaemia	3	No	0	0	0	100	
Gastric	2	No	0	0	0	100	
Pancreas	2	No	0	0	0	100	
Liver	1	No	0	0	0	100	
Oesophagus	1	No	0	0	0	100	
Gallbladder	1	No	0	0	0	100	
Myeloma	1	No	0	0	0	100	
Testes	1	No	0	0	0	100	
Other	1	No	0	0	0	100	
